# Coding for quality? Accountability work in standardised cancer patient pathways (CPPs)

**DOI:** 10.1177/13634593211013882

**Published:** 2021-04-30

**Authors:** Erna Håland, Line Melby

**Affiliations:** Norwegian University of Science and Technology (NTNU), Norway; Sintef, Norway

**Keywords:** accountability, cancer, cancer patient pathways (CPPs), coding, quality

## Abstract

A vital part of standardised care pathways is the possibility to measure performance through different indicators – for example, codes. In this article, based on interviews with health personnel in a project evaluating the introduction of standardised cancer patient pathways (CPPs) in Norway, we explore *the specific types of work involved when health personnel produce codes as (intended) signifiers of quality.* All the types of work are dimensions of what we define as *accountability work* – work health personnel do to make the codes signifiers of quality of care in the CPP.

Codes and coding practices raise questions of what quality of care represents and how it could and should be measured. Informants in our study advocate for coding as important work *for the patient* more than for ‘the system’. This shows how *organising* for quality becomes a crucial part of professional work, expanding what it means to perform high quality care.

## Introduction

Standardisation of care processes, for example through standardised care pathways, represents a common feature of modern healthcare. The intention is to make care processes predictable and safe; to secure care of high quality for all patients. The idea of *accountability* lies at the core of these initiatives. Standardised care pathways are seen as an instrument to make the healthcare system accountable (Martin et al., 2017). This ‘new’ form of accountability implies that trust, to a larger degree, is seen as built into the standardised system, instead of a ‘traditional’ form where trust is directed towards professionals carrying out their work in a satisfactory manner (Jacobsson, 2000; Timmermans and Berg, 2003; Timmermans and Epstein, 2010). On a more overarching level, this development is linked to discussions on how the proliferation of evidence-based medicine and clinical practice guidelines as tools to standardise and monitor clinical practice, challenge professional autonomy, experience and knowledge. As part of these discussions, authors point to that standardisation of care needs to be situated and contextualised (Zuiderent-Jerak, 2007), and that the development of clinical practice guidelines are not neutral, but that they are elements in a chain of translations linking different processes and interests (Knaapen, 2010), drawing upon a diversity of knowledge (Moreira, 2005). In line with these perspectives, we understand standardisation of care processes as social, collective work.

Care pathways are usually connected to, but are not the same as, clinical practice guidelines; they concern the logistics of who should do what and when. Care pathways can be understood as coordinating tools that reconfigure responsibilities, documentation practices and care processes, and they can act as a tool for visible compliance with external regulations (Allen, 2009a; Martin et al., 2017). The degree to which care pathways achieve the promised goal of better quality of care is debated (Allen, 2009b, 2013), and several studies find health professionals resisting standardisation efforts of this kind (Martin et al., 2017). However, for example Martin et al. (2017) also find that health professionals appreciate, adapt to and modify care pathways.

A vital part of many care pathways is the possibility to measure performance through different indicators – for example, codes. These codes can be made available to managers, politicians, the media and the public, implying that clinics and hospitals can be scrutinised and compared, and hence made accountable. Coding are therefore of vital importance in many healthcare systems, making coding practices interesting to study from a sociological point of view, as they play directly into health personnel’s daily work practices. How do health personnel understand and work with coding, and in what ways are codes seen as the representations of quality that they are intended to be? Although many researchers have studied care pathways and their implications for medical practice (see for example Allen, 2009a, 2009b, 2013, 2014; Håland et al., 2015; Røsstad et al., 2015), less is known about the coding processes that are often integrated in these pathways. Coding might appear to be a straightforward and mundane activity, but studies have shown how data and guidelines are created through effortful, situated work carried out by people (Bossen et al., 2019; Knaapen, 2010; Moreira, 2005; Zuiderent-Jerak, 2007), and that the datafication of healthcare require healthcare organizations to re-organize their practices and personnel around data production (Bossen et al., 2019). Bossen et al. (2019) argue that the socio-technical practices of producing and using data have been little investigated and that more research into this area is needed. Aiming to make a contribution in this respect, in this article, based on interviews with health personnel in a project evaluating the introduction of standardised cancer patient pathways (CPPs) in Norway, we explore *the specific types of work involved when health personnel produce codes as (intended) signifiers of quality.*

### Cancer patient pathways (CPPs)

Norway introduced CPPs in 2015, based on current guidelines for cancer diagnostics and treatment. CPPs are national standardised patient pathways which are discipline- and target-based, aiming to minimise waiting times and make cancer care more predictable and safer for patients (The Norwegian Directorate of Health, 2014). The CPPs comprise the period from the presentation of a suspicious cancer symptom until the beginning of cancer treatment and describe steps, for example, referral to specialist healthcare and tests that should be performed in the pathway. One important part of the CPPs are different codes aimed at indicating maximum use of time in the different phases of the pathway. For example, for breast cancer, the maximum timeframe from the suspicion of cancer to the first appointment at the hospital is 7 days, from the first appointment to the clinical decision is 7 days, and from clinical decision to the start of initial treatment is 10–13 days. Health personnel responsible for coding enter specific, standardised codes, usually in the electronic patient record, indicating the start/completion for each phase in the pathway. These codes are comprised in the two main performance goals stated by the Norwegian Directorate of Health: 70% of all cancer patients should be part of a CPP (code OA1), and 70% of all the patients in the CPPs should be taken care of within the indicated maximum timeframes (code OF4). These two codes represent the basis for two of the national quality indicators (NQI) within the Norwegian healthcare system and are made public for each hospital and region on the national website every month. On the basis of these reports, hospitals across the country can be compared regarding compliance to timescales. Compliance is addressed in meetings with managers and the Norwegian directorate of health, and hospitals also sometimes attract attention from the media when they perform ‘bad’ on these indicators. The phases and codes comprised in OF4 can be visualised in this way:

**Figure:**
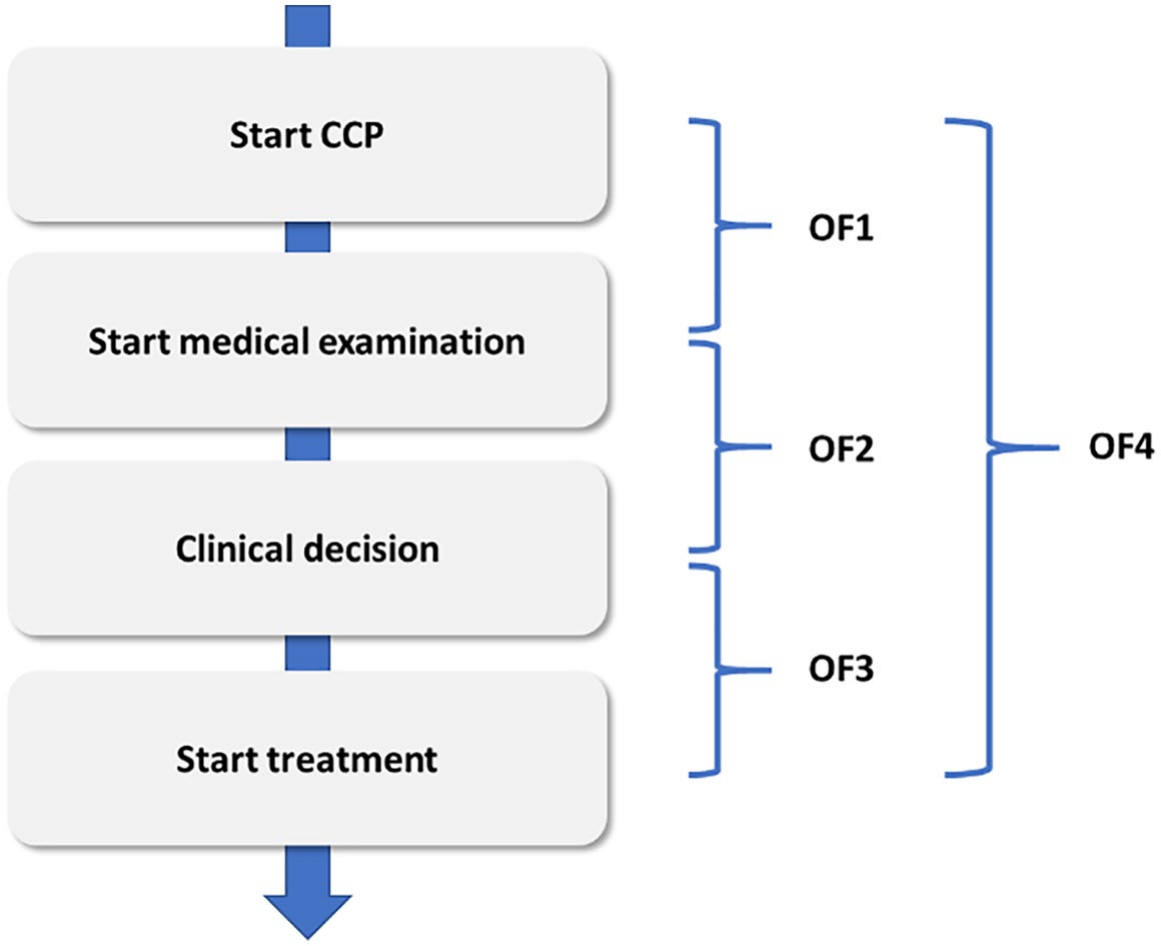


A new position accompanying the introduction of CPPs has been appointed – cancer pathway coordinators. Cancer pathway coordinators is the main group responsible for coding, but other types of health personnel also undertake coding work. There have been no additional resources from the government following the introduction of CPPs; hospitals are expected to handle the introduction within the resources that they already have. This includes the new positions of cancer pathway coordinators. The work has been assigned to existing positions of (mainly) nurses and clerical workers. Thus, coding represents new and additional forms of work for several groups of health personnel, and comprise new challenges and new perspectives for defining what is important work and what is important for quality of care.

## Theoretical framework – accountability and professional work

### Work

Work represents a crucial sociological phenomenon, and has been investigated from many different perspectives. What counts as ‘real’ work depends on the context and who gets to define the work’s meaning (Star and Strauss, 1999). Work is related to the division of labour; how tasks are distributed between actors. These tasks can be imposed, requested, assumed or delegated, and can be accepted or rejected (Strauss, 1985). Some work may be defined as not important, ‘dirty’ work, work that may be a symbol of degradation or something that undermines an individual’s dignity (Hughes, 1958), often resisted by powerful groups in the division of labour. The totality of tasks, the actors and the relations between them need to be articulated and coordinated, and Strauss (1985) labels this work ‘articulation work’. Articulation work is work that “gets things back ‘on track’ in face of the unexpected” (Star and Strauss, 1999: 10). This work is often invisible and not assigned to any specific actor or group of actors, even if this work is considered crucial to get the job done. In this article, we explore the different types of work coding represents when used as signs of quality.

Work and the distribution of tasks are linked to accountability. Actors are responsible for doing their part of the totality of tasks; they are accountable for performing the tasks to certain criteria (Strauss, 1985). These criteria can be subject to discussions and negotiations. Actors are not only accountable for carrying out tasks, but also have the rights to perform tasks and articulate them (Strauss, 1985). These rights are negotiated in relation to other groups in the division of labour, and can be modified and changed, accepted or rejected (Fournier, 2000; Håland, 2012; Strauss, 1985). Importantly, Strauss (1985: 7) points out that ‘accountability requires the work of reporting accountability: tasks involving to whom the actor reports, when, where, how, how much, and even perhaps the necessity of proving that the tasks were done because the acts of carrying them out were invisible to the reportee’. Relevant to our study, the codes in the CPP can thus be seen as ‘proof’ of tasks being undertaken for politicians, authorities and the general public. The reporting of accountability is important for further action and interaction between the different actors (for example between health personnel in the care pathway), and involves work (Strauss, 1985). The codes in the CPPs represent a system of accountability which has direct consequences for the carrying out of work, and it is these types of work that we try to track down.

### Standards and standardisation

Codes can be understood as standardised tools for accountability. Standards and standardisation imply processes of creating uniformity across time and locations (Bowker and Star, 1999; Timmermans and Berg, 1997; Timmermans and Epstein, 2010). The aim of standardisation processes is to make the world easier to grasp, and to avoid chaos and too much variation (for example regarding quality of care). Even though standards do not fully determine action – they can be resisted and modified – they are difficult to ignore (Timmermans and Epstein, 2010). Standards are not neutral, they valorise some perspectives and actions at the expense of others (Bowker and Star, 1999), and different standards generate different outcomes for different users (Timmermans and Epstein, 2010). Health personnel are not passive receivers (or opposers) of standards, as standards and health personnel ‘mutually transform each other during their interactions’ (Timmermans and Berg, 2003a: 76). An important argument made by Timmermans and Epstein (2010) is that standardisation is a social act and standards the product of collective work and effort. Timmermans and Epstein (2010) argue that standardised processes are often more transparent, making them attractive tools for accountability.

### Accountability

Accountability has always been part of professional work, but how this has been performed has varied over time. Traditionally, accountability has been connected to autonomy and self-regulation by the professional community. It has long been argued that autonomy and self-regulation are challenged by cultural, social and political questioning of professional power, and by augmented external regulation (Evetts, 2002; Flynn, 2002). External regulation, for example in the form of clinical practice guidelines, thus represents other forms of accountability (Timmermans and Berg, 2003b). With clinical practice guidelines and care pathways, external parties can measure outcome, compare units and reach control over costs. Accountability means that activities are made visible/transparent to other parties which do not themselves conduct the activities (Triantafillou, 2015). These other parties (e.g. managers, politicians, patients) most often do not have the time or competence to directly observe and understand the activities; they therefore need visualisations, like codes, that simplify them. Also, an important motivation for making services accountable is to be able to govern their conduct (Triantafillou, 2015). Codes as visualisations of quality in cancer care can therefore be powerful tools for managers, health bureaucrats, politicians and others seeking to govern the conduct of health personnel.

### Professional work, autonomy and discretion

There have been many studies exploring how professionals have become subject to, resist and adapt to managerial control/external regulation (see for example Noordegraaf, 2015; Waring, 2007). Noordegraaf (2015) argues for the need to move beyond dualistic and oppositional understandings of professionalism versus managerialism, and claims that quality should no longer be seen as professional property in need of protection from managerial control. Quality is instead seen as consisting of many, different elements, including organisational aspects. Noordegraaf (2015: 2) thus argues that: ‘Not merely *offering* quality when cases are treated, but *organizing* for quality becomes a central ingredient of professional work’. Professions and professional work are dynamic and changing in different contexts, and professionals will have to adapt to changes regarding, for example, new technologies, new expectations of patients, and new ways of collaborating. This might also include, Noordegraaf (2015) argues, new values such as speed. He points to how new values of speed implies that case treatment has to be streamlined in order to act smoothly and quickly, and that these changes affect how professionals act. Speed represents a core value in CPPs, and it is thus interesting to study *how* this affects the way in which professionals act.

Discussions of quality and accountability in professional work also concern professional autonomy and discretion. Evetts (2002) argues that discretion, rather than autonomy, is the most important aspect of professional work (today). Discretion implies using professional judgement to evaluate, advise and perform different forms of action and treatment, but Evetts (2002: 345) highlights how discretion requires the professional ‘to make decisions and recommendations that take *all* factors and requirements into account. These factors will include organisational, economic, social, political and bureaucratic conditions and constraints. Thus, professional decisions will not be *solely* based on the needs of individual clients, but on clients’ needs in the wider corporate, organisational and economic context’. Relevant to our study, Evetts incorporates external regulation of professional performance in the concept of discretion, as opposed to a (more traditional) understanding where external regulation is seen as in opposition to professional discretion.

### Audit

New forms of accountability imply new forms of audit and control. Audit represents a tool for governing at a distance, and Rose (1999: 154) argues that: ‘Rendering something auditable shapes the process that is to be audited: setting objectives, proliferating standardised forms, generating new systems of recordkeeping and accounting, governing paper trails’. Audit is thus concerned with transparent accountability and standardisation, challenging trust in professionals by displacing the basis of expertise by the basis of new norms and objectives (Flynn, 2002; Rose, 1999). Reported codes represent the audit system in the CPPs. Following Rose (1999), this means that coding shapes the process that is to be audited: cancer care itself.

## Methods and material

This study is part of a larger research project evaluating the introduction of CPPs in Norway, funded by the Research Council of Norway (project number 272665). Ethical approval for this study was granted by the Norwegian Centre for Research Data (project number 58724). The empirical material for this particular study is based on interviews with 56 physicians, nurses and cancer pathway coordinators in five hospitals in Norway in the period from 2018–2019, which was three years after the first introduction of CPPs in Norway. The hospitals were chosen to ensure regional diversity across Norway and experiences from both small and large hospitals. All participants received written information about the project prior to interview and signed a consent form. The interviews were conducted by both authors as well as by other researchers participating in the project. All interviews were made available to all researchers in the project, and all interviews were transcribed. The aim of the interviews was to gather information on the participants’ subjective definitions and experiences (Brinkmann, 2018), focusing on how the participants perform and experience CPPs.

The first author conducted the initial analysis of the interviews, and is the main producer of text, discussing both findings and text with the second author. Coding, as a crucial activity within CPPs, emerged as an interesting theme early in the project, both in formal settings (interviews) and informal settings (meetings, conferences). It was striking how a phenomenon which was presented as standardised and straight forward work by the health authorities was discussed and considered problematic by health personnel, initiating a curiosity to investigate what coding represented to health personnel and how it was performed. The data analysis process had the empirical material as the point of departure, searching for and categorizing themes related to how health personnel talk about codes and coding work, moving back and forth between the empirical material and the theoretical concepts in later stages of the analysis.

## Accounting for quality of care in standardised cancer patient pathways (CPPs)

Accounting for quality of care requires a lot of different work from several groups of health personnel in the hospitals. Based on the interviews, we have developed five categories signifying the work involved: *standardisation work*, *legitimisation work*, *jurisdiction work*, *professional discretion work* and *compliance work*. All these types of work are dimensions of what we define as *accountability work* – work that health personnel do to make the codes signifiers of quality of care in the CPP. Implied in this work is also the handling of dilemmas and contradictions, as well as resistance. We investigate each of the types of work below. They are not to be understood as separate and mutually exclusive parts of work, but as different aspects of all the work going on at the same time and performed by different groups of health personnel.

### Standardisation work

Coding work is supposed to be completely standardised, but informants report that there are many different interpretations of which codes should be used when, and many different practices across hospitals and regions. The theme has even been the focus of an experience conference held by the Norwegian Directorate of Health in 2018, and several hospitals and regions have established their own networks and conferences concerning coding practices. These activities, and the experiences made by our informants, indicate that coding are complex practices, and that it requires a lot of different work from health personnel to establish the codes in the CPP as standards. This is in line with how Timmermans and Epstein (2010) argue that standardisation is collective work, and that standards are created by numerous parties coming together to obtain coordination, comparability and compatibility across contexts. Health personnel in our study work to agree upon coding practices that make the published numbers comparable across hospitals and regions.

Some of the health personnel also miss codes which are applicable for the complex world of different patients and different needs, for example codes for patients who want to postpone the start of their initial treatment due to planned vacations or family events. When there are no codes for this, the hospital gets ‘bad results’, even if the postponed treatment is requested by the patient and medically safe. Several of these concerns have been brought to the Norwegian Directorate of Health’s attention, and there are codes currently being developed for some of these events. This development also shows how codes and coding practices are dynamic and negotiable, how standards are developed by many parties (Timmermans and Epstein, 2010) and how standards and health personnel mutually change each other through interactions over time (Timmermans and Berg, 2003a).

Some of the challenges with establishing comparable codes as standards, as described above, are expressed by one of the cancer pathway coordinators (CPCs) in this way:

CPC: We just had a case, we received a call from a newspaper, where they were comparing times in the breast cancer pathway, and we got a bad result. It is really difficult, because you do not have the same perception as the numbers show, and I feel that I know why the numbers are as they are, and that could be because I have not had the time to ‘after-code’ [not code in real time].

Interviewer: So, simple things like that actually?

CPC: Yes. And another thing is that I know that another hospital interprets the pathway (codes) completely differently from us, and has a different practice. . .for example, I know that now I have had three to four patients who wanted to go on vacation before surgery, so I do not think that this is. . .it is a good tool to, in a way, partly see the statistics, but at the same time you need to know what it is that you are seeing, and you have to know what kind of eventualities that can influence this. (CPC 1, hospital 2)

This quotation shows how a ‘standardised’ number, in this case, is not standardised, and needs to be interpreted within a larger context. The visible sign of accountability (Strauss, 1985; Timmermans and Berg, 2003b; Triantafillou, 2015) (the ‘good’ number) is not there, therefore the hospital gets attention from the media over ‘bad results’. The coordinator has plausible explanations for why the numbers are ‘bad’, but the audit system challenges trust in her as a professional by replacing the basis of expertise with the basis of other norms and objectives (Flynn, 2002; Rose 1999). Furthermore, this quotation also shows how standardisation valorises some values at the expense of others (Bowker and Star, 1999). The value of speed is valorised at the expense of the value of patients’ individual preferences (vacation before surgery).

The informants describe the importance of sharing experiences in order to establish the same practices. The challenges of coding practices being open for interpretation and the collective work (Timmermans and Epstein, 2010) involved in making coding standardised practices are described in this way by one of the coordinators:

The guidelines from the Directorate of Health give room for different interpretations, and we were one of the first starting with the pathway and we have had a lot of contact with the Directorate about how to code this, how to code that, what do you want, but it has been. . .a little so and so. We have received a lot of feedback, but we see that it varies a lot how we solve this [. . .] today we are very. . .what are we doing? Is it useful? Are you using this for anything? And since it gives room for interpretations to that extent, then it is done differently, so we ourselves have asked for a network for cancer pathway coordinators, in this hospital, and we have started one. So, once a month we have a meeting with all the coordinators in the hospital where we can share experiences, different problems, what do you think of this, that has been really ok. (CPC 1, hospital 2)

The coordinator expresses how they themselves have felt the need to share experiences, and have requested more information from the Directorate of Health, but have established their own network addressing these issues. This quotation also shows how standards – and *the outcome* of standards – needs attention over time. The coordinator describes how, three years after the implementation, they ask for results – is it meaningful to keep coding, what purpose does it serve?

Standardisation work implies health personnel creating networks and arenas to interpret, discuss and share experiences of coding in order to establish comparable and meaningful coding practices. Included in these discussions are also questions addressing the very purpose of codes and coding as signifiers of quality.

### Legitimisation work

Health personnel who do the coding, work to legitimise coding as being important and make their colleagues accept this as important numbers and important work – to define it as ‘real’ work (Star and Strauss, 1999). Some health personnel know little of the coding process, care little about the numbers and express that the codes are just there for the bureaucracy and have little to do with what counts as important in clinical work.

One of the physicians says:

It is a parallel universe with codes and registrations. We clinicians have a real perception of what is urgent and what is not, and then we do the job anyway. Melanoma is a serious diagnosis which should be prioritised no matter what and it has always been like this. (Physician 2, hospital 2)

The physician expresses how they prioritise cancer as they have always done, and that the codes are completely separated from this clinical work, as she sees it – they exist in a ‘parallel universe’. Coding, from her perspective, can thus be understood as work that is not important, showing that what counts as ‘real’ work depends on the context and who gets to define what counts as important work (Star and Strauss, 1999).

Other health personnel, especially the coordinators, work to legitimise codes as important both for the follow-up of the patients and for the reputation of the hospital. Two of the coordinators express it in this way:

The point is that coding is extremely important in the follow-up of the patient, so that I think that the closer it [the coding] takes place to the person who actually talks to the patient and is responsible for the follow-up, the better. (CPC 3, hospital 4)

I think that it means a lot, even if you do not have this patient contact, it is a human being who sits there and keeps track and do one’s best to get the patients in. So, I noticed myself, in that position, I got very concerned with these timescales [codes] [. . .] One gets dedicated to achieve this, to get everybody in [included in the pathway], so in my head, 70% was not a target figure for me, I want 100%, so I have tried to include as many as possible. Very fun, useful work. (CPC 2, hospital 3)

The coordinators clearly see coding as meaningful work, and as work performed from a motivation to do one’s best for *the patients*, more than ‘for the system’. We argue that by defining coding work as important for the patient, instead of as important for some vague and abstract notion of a ‘system’, coding is made legitimate to health personnel with patient work as a core part of their professional identity. Also, one of the coordinators argues that the coding should be performed by someone close to the patient, who knows the patient’s follow-up process. This could be understood as going against an understanding of coding as something bureaucratic, which could be performed by anyone skilled in administrative work and separate from the patient. It can be argued, from the point of view of this coordinator and other health personnel in our study, that coding *is* – legitimate – patient work. This is in line with how Noordegraaf (2015) argues that *organising for quality* is an important part of professional work.

This understanding is, however, not shared by everyone. Some of the health personnel, also the coordinators performing the actual coding, question codes as signs of quality:

I think it is a little two-fold, I think that it is a good thing for the patients, but I do not think that it necessarily says something about the treatment that has been given, because if you fail to code one, then it is like it [the patient] has not been given the treatment it was supposed to have, but it is perfectly possible that it has. At the same time, we are being measured on this, so we just have to relate to that. (Nurse 1, hospital 2)

The nurse points to the fact that coding does not necessarily indicate a meaningful representation of quality of care. The patient may have received proper treatment at the right time in the pathway, but if the coding is not there, the visible sign of accountability is not present (Strauss, 1985; Timmermans and Berg, 2003b; Triantafillou, 2015). This can be understood as ‘trust in the system/numbers’ instead of trust in professionals (Timmermans and Berg, 2003; Timmermans and Epstein, 2010; Jacobsson, 2000).

Legitimisation work implies health personnel working to define coding as important work. For health personnel recognising coding as important, and with coding as part of their work, a lot of work is performed in order to make other health personnel accept this as important work and to make other health personnel understand how the work they do affects the coding work. In general, we find that health personnel seeing coding as important work defines the importance in relation to what benefits *the patients* more than what benefits the hospital/system.

### Jurisdiction work

There is some diversity across groups of health personnel, hospitals and regions in our empirical material concerning *who* should perform the coding. Most of the coding is performed by coordinators or other administrative staff. Some of the coordinators are nurses, while others are clerical workers. In some hospitals, coding represents a small part of the coordinators’ work, while in other hospitals there are staff who are assigned to coding only. Physicians also do (or should do) some parts of the coding, and the coding process is dependent on the different parts being performed in order to achieve the main parameter, the total timespan to initial treatment (OF4). There are also discussions about who should have the final word in deciding to start the CPP, and who should decide which code to set when. Actors’ rights to perform and articulate tasks in the division of labour are subject to negotiations (Fournier, 2000; Håland, 2012; Strauss, 1985), and we see negotiations between different groups and professions regarding coding practices.

A coordinator expresses how there are sometimes challenges with physicians not performing coding, even though they are supposed to, and how he takes the responsibility for coding:

Then I have to read the referral well, what this is about, and then one puts CPP or not [code for start CPP]. It is actually the physician who should set CPP, right, but they do that very rarely and when they do it, they can do it wrong. So, I often go through all the referrals to be sure that as many [patients] as possible are included. (CPC 1, hospital 4)

The coordinator explains how he has to read the referral properly, to decide whether it is CPP or not. This is supposed to be the physicians’ work; it lies within the physician’s jurisdiction, but they do not do it or they do it ‘wrong’. The coordinator therefore has to do the physicians’ work. This quotation illustrates how the coordinators have gained the right to decide whether they should start a CPP or not, even though they have no medical background for making these decisions and it lies within the physicians’ jurisdiction. The hospitals have made these decisions in order to make the system or the coding process work, as the physicians simply do not do what they are supposed to do. This shows how the rights to perform tasks are negotiated between actors, and can be assigned (the cancer pathway coordinators) or rejected (the physicians) (Fournier, 2000; Håland, 2012; Strauss, 1985). Also, this can be understood as physicians resisting what they define as ‘administrative work’, or which is understood to be ‘dirty’ work degrading to their professional identity (Håland, 2012; Hughes, 1958). Some of the nurses and coordinators also describe a work practice where they facilitate the physicians’ coding.

The resistance towards coding as part of the physicians’ work is expressed by several of the physicians themselves, for example:

It should not be the physicians’ job to do this coding, when it is not automated. We can be asked when the clerical workers are in doubt; the clerical workers need to be able to read the medical records, and understand what it says, but it is my strong opinion that physicians should not be made to do coding work. (Physician 2, hospital 2)

The physician describes that coding should not be part of the physicians’ work. This can be seen as a rather common way of resisting clerical/administrative work by the physicians, in line with what Hughes (1958) describes as resisting ‘dirty’ work.

There are also discussions between health personnel in the hospital over who should make the final decision about including the patient in the CPP or not when patients are referred from GPs. Should they follow the CPP-code set by the GP or should the hospital make the final decision? One of the physicians describes this practice, making it clear that it is she who decides whether it is a valid referral for CPP or if it is ‘rubbish’:

It is often that they [GPs] send referrals to us marked with ‘CPP’ and some of those referrals are in such a state that we think that this is not a CPP, so then we override that and say that this is just rubbish; it is not something that is supposed to get in [as CPP]. (Physician 1, hospital 2)

Health personnel do jurisdiction work in order to decide who should do what and when in the coding process, negotiating rights in the division of labour (Strauss, 1985). Often, coordinators end up facilitating or doing the physicians’ work, and the physicians themselves often firmly resist coding as part of their work. However, the physicians in the hospitals demand the final word regarding coding as CPP or not, sometimes refusing to code the GPs’ referral as start CPP, thus claiming this as their jurisdiction.

### Professional discretion work

Health personnel work to prioritise time spent on the different needs within the CPP, typically when they should use their time to code and when they should use their time to do other types of clinical work. According to the Directorate of Health, coding should happen in ‘real-time’ and not be conducted later in the process (‘after-coding’). This means that when the patient is admitted, the code should be set for that, when the treatment starts, the code should be set for that etc. When the codes are not set in ‘real-time’, the published numbers every month do not necessarily give the correct impression. However, health personnel report that it is almost impossible to adhere to this, and it varies across hospitals and regions to what extent they do ‘after-coding’. However, they do not express that ‘after-coding’ is an oppression against the system; they simply do not have enough time so they have to use their professional discretion to prioritise between different important tasks.

Health personnel describe that, in a hectic work environment, coding is not always prioritised in real-time; ‘real’ work with patients is considered more important:

I just think that there is a lot of attention to the coding, and it is time consuming and it should be performed correctly; it should just get done on top of everything else that we are supposed to do. It is clear, if you have the choice, to get a patient in within the timescale, then you use the time to plan the patient treatment and maybe not so much the coding in your work day, when you have to prioritise. Therefore, there is some pressure there, but it is easy to choose the patient over coding. But we have to do both, so try to find a balance there, then. (Nurse 1, hospital 2)

This nurse describes that they perceive what is important for the patient so that they are taken care of by the right people at the right times in the pathway. Thus, when they need to prioritise their time, they prioritise planning the patient treatment instead of coding real-time. This shows that they are taking different factors and requirements, including organisational, bureaucratic and political factors, into account when they use their professional discretion (Evetts, 2002), deciding how to prioritise. The same prioritisation is found in the quotation below, also describing the struggle between different priorities:

CPC: No, I have not had anything to do with coding in four months, I just have not had the time. So, I am behind [. . .] and because of that we are not in a good place now, our numbers are very bad at the moment.

Interviewer: But that is because you are bad at coding, to put it that way, not necessarily bad in taking people in for treatment?

CPC: Yes, it is just that. In our last meeting in the clinic, then I just had to say ‘sorry, I am behind with the coding’.

Interviewer: Is it something you do not prioritise if you have to choose between things?

CPC: Yes, my first priority is to get the patients in, it is, so most of the patients get the code we start CPP with, we start the CPP, but then, often, I do not get them out again [laughs] (CPC 3, hospital 2)

The coordinator expresses how she has done the job properly in the sense that the patient has been taken care of, has obtained the required appointments and has been discharged. However, when the coding has not been done, it is not visible. The visible signs of accountability are not there (Strauss, 1985; Timmermans and Berg, 2003b; Triantafillou, 2015). This can be understood as a situation where the audit system implies that trust is built into the system instead of having trust in professionals doing their job properly (Jacobsson, 2000; Rose, 1999; Timmermans and Berg, 2003; Timmermans and Epstein, 2010). However, despite feeling stressed for not doing the coding work in time, health personnel use their professional discretion to always prioritise ‘the patient’, much in line with how taking care of patients can be understood as a core part of the professional identity. Importantly, organisational, bureaucratic and political factors are part of these judgements (Evetts, 2002).

Coding is complex and time-consuming work, and health personnel use their professional discretion to prioritise between coding and other types of work. They have an understanding of ‘getting the patient through’ as the most important part of their work, and they prioritise this at the expense of coding in ‘real-time’.

### Compliance work

Health personnel also do work which they describe as work ‘for the system’, work that they do not really see the meaning of, but which they experience they still have to perform. Our informants express how they take part in these practices to ‘please the system’, or that they refuse to take part, even if this could mean the hospital/the clinic gets ‘bad results’.

Some of the informants describe work that is performed only to make the hospital/clinic ‘look good’, for example regarding ‘after-coding’:

CPC: So, when I started, we had just, I suddenly found out, when we first got a report in this system, we had 30% in CPP and it was supposed to be 70%, right, and then I understood that something must be wrong [. . .] It is supposed to be 70% [one of the main performance goals] and we had 30%–40% and that was a very bad inclusion in the CPP. And then it turned out that there was a mistake, so then we were supposed to do the ‘after-coding’ [. . .]. It was completely ridiculous, because to ‘after-code’ patients that have already gone through this, I mean, that is just for the sake of appearances, it is not for. . .

Interviewer: But did you do it?

CPC: Yes, we started to do it, and then there was some back and forth about whether it should be done, but the clinic wanted it to be done because the numbers looked bad. (CPC 3, hospital 3)

The coordinator describes how they had to ‘after-code’ because of some initial error with the coding. This work had no meaning to them, as the patients had been taken care of, but the coding was not performed as it should have been, so they had to do that work in order to make the clinic look better.

On other occasions, informants describe not wanting to ‘give in’ to the system and making their own prioritisations despite getting ‘bad results’. Health personnel also express that ‘other hospitals’ code things differently (or ‘wrong’), which means that they get ‘good results’ (implied in this is an understanding of other hospitals getting better results than they ‘deserve’). This is described as a major problem when comparing the publicly available numbers across hospitals, as they are not experienced as comparable. Some of the health personnel resist these practices:

I also think that it is a little different how the different [hospitals] start CPP, so that maybe they win a few days and get it really well, but what I think is important, personally I do not care so much about that, because what is important is that we do this properly, so that we can do improvement work. It is no use, kind of, cheating your way to something, so that. . .the coding practices are different; I have seen that around. (Physician 2, hospital 4)

The physician expresses that they should not ‘cheat’ in order to get good results, and that they should do the work properly in order to improve the health services.

Health personnel do compliance work in order to ‘please the system’ for various reasons, even though they feel that it is not meaningful, does not say anything about quality of care and does not have any consequences for patients. When accountability becomes so closely linked to what is *visible* to other parties (Triantafillou, 2015), strategies to comply with the system might be expected. Sometimes they also resist compliance, despite knowing that this will lead to ‘bad results’.

## Concluding remarks

In this article, we have explored the specific types of work health personnel perform when they produce codes as signifiers of quality of care in the CPP. All these types of work are dimensions of what we label *accountability work*. *Standardisation work* is carried out by many parties to try to establish a unified way to code – and a unified outcome of coding – in line with how Timmermans and Epstein (2010) describe standardisation as collective work. The standardised way to code is not there automatically, it has to be negotiated and established across groups and sites – and also in discussions with the health authorities. *Legitimation work* implies work health personnel do in order to legitimise coding as important work. Since what counts as ‘real’ work depends on the context and who gets to define the work’s meaning (Star and Strauss, 1999), the health authorities’ attention to codes does not in itself establish coding as important work. Health personnel who perform the coding have to negotiate, discuss and advocate for coding as important – and some health personnel still do not regard codes as important for quality of care. *Jurisdiction* work is performed to negotiate who should do what and when regarding the coding in the CPP. The cancer pathway coordinators have gained the rights to decide whether they should start the CPP or not, even though they have no medical background for making these decisions and it is the physicians’ jurisdiction. The physicians argue that coding should not be part of their work, even though they claim the right to overrule decisions to start CPPs coming from the GPs. Rights to perform tasks are thus negotiated between actors, and can be assigned (the cancer pathway coordinators) or rejected (the physicians) (Fournier, 2000; Håland, 2012; Strauss, 1985). *Professional discretion work* concerns the work that health personnel do, taking *different* factors and requirements into account when using their professional discretion (Evetts, 2002) to prioritise time spent on different tasks. More specifically, when pressured for time, they prioritise ‘getting the patient through’ over coding work. Finally, health personnel describe how they sometimes do work only to ‘please the system’, which we label *compliance work*. This represents work that they see no meaning in and perform only to make the hospital/clinic ‘look good’. When accountability becomes so closely linked to that which is *visible* to other parties (Triantafillou, 2015), compliance work can be expected to be part of accountability work.

Codes and coding practices raise questions regarding what quality of care represents and how it could/should be measured. Even though several of the informants in our study resist codes as signifiers of quality, many also advocate for coding as important work. More specifically, they advocate for coding as important work *for the patient* more than for some vague notion of ‘the system’. Thus, coding is made legitimate to health personnel with patient work as a core part of their professional identity. Also, this shows how *organising* for quality becomes a crucial part of professional work (Noordegraaf, 2015), expanding what it means to perform high quality care. With this perspective, coding is *part of* professional work, and not some unimportant practice taking place *besides* professional work.

Limitations of our study include that we investigated coding practices early after the introduction of CPPs. Coding experiences and practices may change over time, so our findings must be interpreted with that in mind. Also, we could have benefited from additional observational data of work practices, providing more detailed insight into the actual coding work.

Standards elevate some values and actions at the expense of others (Bowker and Star, 1999). Codes and coding practices as (efforts to be) standardised signifiers of quality – visible signs of accountability (Triantafillou, 2015) – thus elevates some values of quality over others. The visible sign of achieving quality is represented in the two performance indicators (staying within the maximum timespan in the CPP and including a large enough number of patients in the CPP). The crucial question then becomes, does this represent quality in a good way? Which aspects of quality of care are lost/downplayed when these indicators are put to the foreground? We have shown how coding represents a lot of work to many groups of health personnel – is it worth the effort? Within this perspective, the question becomes *for whom* is it meaningful to keep coding and *whose* purpose does it serve? Based on our findings we suggest that implications for practice should be that health authorities reflect upon how and when to use codes as quality indicators and if they serve the purposes they are intended to. With these questions in mind, it is important to further explore and investigate coding and other forms of *organising for quality* as core parts of modern healthcare, and the possible implications this has for perceptions of, and performance of, quality and accountability.
